# Estimation of a Simple Structure in a Multidimensional IRT Model Using Structure Regularization

**DOI:** 10.3390/e26010044

**Published:** 2023-12-31

**Authors:** Ryosuke Shimmura, Joe Suzuki

**Affiliations:** Graduate School of Engineer Science, Osaka University, Toyonaka 560-0043, Japan; prof.joe.suzuki@gmail.com

**Keywords:** prenet penalty, lasso, simple structure, stochastic EM algorithm

## Abstract

We develop a method for estimating a simple matrix for a multidimensional item response theory model. Our proposed method allows each test item to correspond to a single latent trait, making the results easier to interpret. It also enables clustering of test items based on their corresponding latent traits. The basic idea of our approach is to use the prenet (product-based elastic net) penalty, as proposed in factor analysis. For optimization, we show that combining stochastic EM algorithms, proximal gradient methods, and coordinate descent methods efficiently yields solutions. Furthermore, our numerical experiments demonstrate its effectiveness, especially in cases where the number of test subjects is small, compared to methods using the existing $L_{1}$ penalty.

## 1. Introduction

Item Response Theory (IRT) is a mathematical model used for applying and evaluating tests, and is employed in the creation and operation of various large-scale ability tests such as language proficiency exams. Although IRT models are practical, they assume a unidimensional latent trait, which is not suitable when the test measures multiple abilities. Therefore, to measure multiple latent traits, Multidimensional IRT (MIRT) models [1] are utilized, extending the IRT model to multiple dimensions. However, MIRT models can be challenging to interpret from the estimation results, such as understanding what each latent trait represents and the relationships among test items. Therefore, to facilitate interpretation, such as “what latent traits are the test items measuring,” it is desirable to have a simple structure in the estimated matrix, like having many zeros. In this paper, we propose a method for estimating a matrix with a simple structure where one item corresponds to one latent trait, using binary (correct/incorrect) response data.

In MIRT, the simplicity of the estimation results plays an important role in interpretability. Existing research has proposed penalization methods using $L_{1}$-regularization, as employed in lasso [2,3], for MIRT [4]. Using $L_{1}$-regularization shrinks the estimates towards zero, allowing some variables to be precisely zero. Thus, the method simplifies the estimated matrix by excluding unnecessary variables and performing variable selection. The properties of $L_{1}$-regularization in linear regression have been widely studied and are known to provide high-accuracy estimates with consistency in model selection [5,6,7].

However, $L_{1}$-regularization does not necessarily produce interpretable and simple matrices as estimation results. For example, if the regularization parameter is too large, all variables are estimated as zero, making the analysis meaningless. Indeed, numerical experiments (Section 4) have shown that when the number of subjects (examinees) is small, selecting the regularization parameter using the Bayesian Information Criterion (BIC) [8] leads to selecting a matrix where all components are zero. Also, $L_{1}$-regularization uniformly shrinks all variables to zero, leading to more frequent zero estimates for variables close to zero.

In this study, we employ the product-based elastic net penalty (prenet penalty) [9], proposed in the field of factor analysis, for structure regularization. The prenet penalty is a penalty for the product of pairs in the same row of a matrix, and using this penalty ensures that the estimates have at most one nonzero component per row. Therefore, it allows clustering of test items by latent traits, with one item corresponding to one latent trait. If responses are multi-valued, the obtained responses can be treated as continuous values and solved within the framework of factor analysis. However, in cases like this study where responses are binary, solving within the conventional factor analysis framework is unnatural. Thus, this study can be seen as an extension of the prenet penalty to discrete factor analysis. The optimization of the proposed method efficiently combines the stochastic EM algorithm [10], the proximal gradient method [11], and the coordinate descent method [12].

The regularization parameter of the prenet penalty controls the simplicity of the estimated matrix. In this study, the regularization parameter is determined using BIC. Furthermore, a Monte Carlo simulation using synthetic data is conducted to compare $L_{1}$-regularization with the proposed method using prenet. The proposed method with prenet demonstrates its ability to estimate the true structure of the matrix even with a small number of subjects.

The remainder of this paper is organized as follows: Section 2 describes the MIRT model and the prenet penalty dealt with in this study. Section 3 presents the optimization methods to obtain solutions to the proposed method. Section 4 demonstrates the performance comparison between $L_{1}$-regularization and prenet penalty using Monte Carlo simulations with synthetic data. Finally, Section 5 concludes the paper and discusses future extensions.

## 2. MIRT Model and Prenet Penalty

### 2.1. 2-Parameter Multidimensional IRT Model

Consider a situation with responses from *N* subjects to *J* items. The response of subject *i* to item *j* is binary, denoted by $y_{ij}\in \left\{0,1\right\}$. Each subject has *K*-dimensional latent traits, represented by $\theta_{i}\in R^{K},\left(i=1,\ldots ,N\right)$. Assuming that $y_{ij}$ comes from a 2-parameter multidimensional IRT (2-PL MIRT) model with $a_{j}\in R^{K},b_{j}\in R;\left(j=1,\ldots ,J\right)$, the model is

(1) $$\begin{array}{cc} p(y_{ij}=1|\theta_{i},a_{j},b_{j}) & =\frac{\exp(a_{j}^{T}\theta_{i}+b_{j})}{1+\exp(a_{j}^{T}\theta_{i}+b_{j})}\;. \end{array}$$

Furthermore, we assume local independence among responses, that is, let $y_{i}={\left(y_{i1},\ldots ,y_{iJ}\right)}^{T}$, $A={\left(a_{1},\ldots ,a_{J}\right)}^{T}$, and $b={\left(b_{1},\ldots ,b_{J}\right)}^{T}$. Then, it is assumed that

$$p\left(y_{i}|\theta_{i},A,b\right)=\prod_{j=1}^{J}p\left(y_{ij}|\theta_{i},a_{j},b_{j}\right)$$

holds.

Assuming the prior distribution of $\theta_{i}$ as ϕ, the likelihood function for the complete data, given the latent traits $\Theta ={\left(\theta_{1},\ldots ,\theta_{N}\right)}^{T}$ and responses of each subject $Y={\left(y_{1},\ldots ,y_{N}\right)}^{T}$, is

(2) $$\begin{array}{c} L\left(A,b|Y,\Theta \right)=\prod_{i=1}^{N}\phi \left(\theta_{i}\right)\prod_{j=1}^{J}p\left(y_{ij}|\theta_{i},a_{j},b_{j}\right) \end{array}$$

In this paper, ϕ is modeled as the density function of independent normal distributions $N(0,I_{K})$, where $I_{K}$ is the *K*-dimensional identity matrix. While it is possible to estimate the covariance matrix Σ by considering a prior distribution of N(0,Σ), in this context, we choose to fix Σ as $I_{K}$. The log-likelihood of Y, marginalized over Θ, is

(3) $$\begin{array}{c} \ell \left(A,b|Y\right)=\sum_{i=1}^{N}\log\left(\int \phi \left(\theta_{i}\right)\prod_{j=1}^{J}p\left(y_{ij}|\theta_{i},a_{j},b_{j}\right)d\theta_{i}\right) \end{array}$$

In marginal maximum likelihood estimation of conventional MIRT, one seeks to maximize (3) to find A,b. However, this study considers adding regularization *P* to impose a simple structure on A, with ρ>0 as the regularization parameter:

(4) $$\begin{array}{c} \left(\hat{A},\hat{b}\right)=\underset{\left(A,b\right)}{\mathrm{argmax}}\left\{\frac{1}{N}\ell \left(A,b|Y\right)-\rho P\left(A\right)\right\} \end{array}$$

Setting $P\left(A\right)={∥A∥}_{1}=\sum_{j=1}^{J}\sum_{k=1}^{K}\left|a_{jk}\right|$ results in $L_{1}$-regularization, as proposed in [4].

### 2.2. Item Clustering Using structure Regularization

With $L_{1}$-regularization like in lasso [3], when ρ→∞, the estimate $\hat{A}=O$, making clustering infeasible. In addition, the estimates are reduced by ρ. This study uses γ∈[0,1] for the product-based elastic net penalty (prenet penalty) [9]:

(5) $$\begin{array}{cc} P(A) & =\sum_{j=1}^{J}\sum_{k=1}^{K-1}\sum_{k^{\prime }>k}^{K}\left\{\gamma |a_{jk}\left|\right|a_{jk^{\prime }}|+\frac{1}{2}\left(1-\gamma \right){\left(a_{jk}\right)}^{2}{\left(a_{jk^{\prime }}\right)}^{2}\right\}\\ A & ={\left(a_{1},\cdots ,a_{J}\right)}^{T}=\left(\begin{array}{ccccc} a_{11} & \; & \cdots & \; & a_{1K}\\ \vdots & \; & \ddots & \; & \vdots \\ a_{J1} & \; & \cdots & \; & a_{JK} \end{array}\right) \end{array}$$

**Figure 1:**
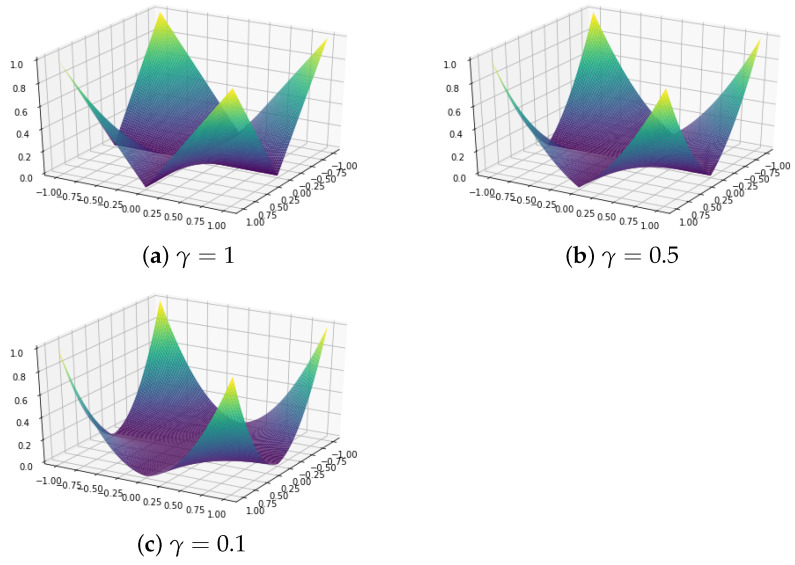
The prenet penalty for γ=1,0.5,0.1.

The prenet penalty ($P\left(x,y\right)=\gamma |x||y|+\left(1-\gamma \right)x^{2}y^{2}/2$) for γ=1,0.5,0.1 is shown in Figure 1. Prenet has a pointed shape when x=0 or y=0, and although it is not a convex function overall, it has the property of being multi-convex, becoming convex when other variables are fixed. Due to this property, an efficient solution can be obtained using the coordinate descent method.

**Figure 2:**
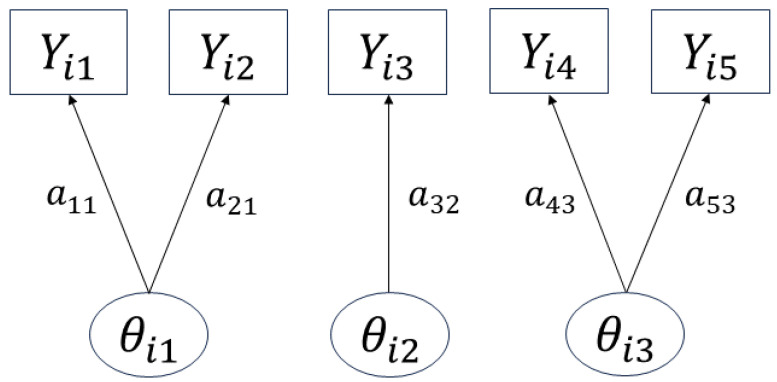
Image of the obtained estimation results.

Using the prenet penalty, when ρ→∞, a simple structure is imposed on $\hat{A}$. In fact, when γ∈(0,1], as ρ→∞, $\hat{A}$ has at most one nonzero component per row (Proposition 1 in [9]), thus leading to a situation like in Figure 2, where each item corresponds to at most one latent trait. Therefore, it becomes possible to categorize each item by the necessary latent traits, enabling item clustering. The prenet penalty is also discussed in relation to k-means in [9], and can be seen as a generalization of the k-means.

### 2.3. Determining the Regularization Parameter ρ

In this study, the regularization parameter ρ is determined using the Bayesian Information Criterion (BIC) [8]. The BIC is defined as follows:

(6) $$\begin{array}{c} BIC=-2\ell \left(A,b|Y\right)+p_{0}\log N \end{array}$$

Here, $p_{0}$ represents the number of nonzero components in *A*. The BIC applies a penalty based on the number of nonzero components in A, thus penalizing the model’s complexity. We calculate the BIC (6) for several values of ρ and select the parameter that minimizes the BIC. The parameter γ∈[0,1] for the prenet penalty should also be determined using the BIC, but in the experiments of this paper, only γ=0.1 is used. Furthermore, when clustering is the goal, a sufficiently large ρ can yield item clusters, but if ρ is too large, the non-convexity of the prenet penalty becomes strong, leading to convergence to a local minimum and instability in the solution. Therefore, it is advisable not to make ρ excessively large.

Just as in factor analysis, when the identifiability conditions of the orthogonal model (e.g., Theorem 5.1 in [13]) are satisfied, the solution becomes unique apart from the indeterminacy of orthogonal rotation. To fix this rotation, some constraints must be imposed on *A*. For instance, $a_{jk}=0$ for j=1,…,K, k=j+1,…,K−1. Under these identifiability conditions and the constraint that A has at most one nonzero component per row, implying the perfect simple structure, A becomes unique except for the sign and permutation of columns. Using the prenet penalty, A with the perfect simple structure can be estimated when ρ→∞. Therefore, in this study, we conducted estimation without adding any special constraints other than the prenet penalty.

## 3. Optimization Method

This section discusses methods to solve the optimization problem (4). In this paper, we employ the stochastic expectation-maximization (stEM) algorithm, as proposed in standard marginal likelihood estimation [10,14]. The EM algorithm is a method that seeks a solution by repeating the E-step, which calculates the expected log-likelihood of the posterior distribution, and the M-step, which maximizes the expected log-likelihood obtained in the E-step. In the stEM algorithm, the E-step is efficiently performed using random numbers from the posterior distribution, consequently making the calculations in the M-step more efficient.

### 3.1. Stochastic E-step (stE-step)

Let $\left(A^{\left(t\right)},b^{\left(t\right)}\right)$ be the parameters at the *t*-th iteration. In the standard E-step, for iteration t+1, the expected log-likelihood of the posterior distribution is computed as follows:

(7) $$\begin{array}{c} Q\left(A,b|A^{\left(t\right)},b^{\left(t\right)}\right):=E_{\Theta }\left[\frac{1}{N}\sum_{i=1}^{N}\log\left(p\left(y_{i}|\theta_{i},A,b\right)p\left(\theta_{i}\right)\right)|Y,A^{\left(t\right)},b^{\left(t\right)}\right] \end{array}$$

In the case of MIRT model, it is difficult to compute this as usual, so existing research [4] has used a lattice point approximation. In this study, we approximate by generating random numbers from the posterior distribution using the Markov chain Monte Carlo (MCMC) method, namely Gibbs sampling. The details of the random number generation method using Gibbs sampling are described in Section 3.3.

In the StE-step, random numbers from the posterior distribution are generated only once per iteration. That is,

(8) $$\begin{array}{c} {\tilde{\theta }}_{i}^{\left(t+1\right)}∼p\left({\tilde{\theta }}_{i}^{\left(t+1\right)}|y_{i},A^{\left(t\right)},b^{\left(t\right)}\right) \end{array}$$

is sampled, and

(9) $$\begin{array}{c} Q\left(A,b|A^{\left(t\right)},b^{\left(t\right)}\right)\approx \frac{1}{N}\sum_{i=1}^{N}\log\left(p\left(y_{i}|{\tilde{\theta }}_{i}^{\left(t+1\right)},A,b\right)p\left({\tilde{\theta }}_{i}^{\left(t+1\right)}\right)\right) \end{array}$$

is approximated in this way.

### 3.2. M-Step

In the M-step, we maximize the regularized expected log-likelihood obtained in the stE-step (9). Specifically, using ${\tilde{\theta }}_{i}^{\left(t+1\right)}$ as the random numbers generated in the stE-step, we solve for

(10) $$\begin{array}{c} \left(A^{\left(t+1\right)},b^{\left(t+1\right)}\right)=\underset{\left(A,b\right)}{\mathrm{argmax}}\left\{\frac{1}{N}\sum_{i=1}^{N}\log\left(p(y_{i}|{\tilde{\theta }}_{i}^{\left(t+1\right)},A,b)\right)-\rho P\left(A\right)\right\}. \end{array}$$

The part to be maximized, the expected log-likelihood, is composed of *J* logistic regressions, allowing easy calculation of gradients and Hessians. Therefore, it can be calculated using methods such as the proximal Newton method [15]. In this paper, we solve it using the proximal gradient method [11] and the coordinate descent method for $L_{1}$-regularization [12]. Details of the optimization calculations are presented in Appendix A.

### 3.3. Gibbs Sampling

We consider the method of sampling from the posterior distribution in the StE-step. In this study, following [16], we generate random numbers from the posterior distribution using Gibbs sampling with the Pólya-Gamma distribution. This approach is an extension of the method proposed for logistic regression [17].

**Definition** **1.** *When a random variable X follows the distribution*

(11) $$\begin{array}{c} X∼\sum_{k=1}^{\infty }\frac{G(b,1)}{2\pi^{2}{\left(k-0.5\right)}^{2}+c^{2}/2} \end{array}$$

*we say that X follows the Pólya-Gamma distribution with parameters b>0,c∈R, denoted as X∼PG(b,c). Here, G(b,1) is the gamma distribution with parameters b,1.*

Using the Pólya-Gamma distribution, the logistic function for ψ∈R,a∈{0,1} can be expressed as an integral in the following form:

(12) $$\begin{array}{c} \frac{{\left(\exp\left(\psi \right)\right)}^{a}}{1+\exp(\psi )}=2^{-1}\exp\left(\kappa \psi \right)\int_{0}^{\infty }\exp\left(-\frac{w\psi^{2}}{2}\right)p\left(w|b,0\right)dw \end{array}$$

where p(w∣b,0) is the probability density function of PG(b,0) and $\kappa =a-\frac{1}{2}$. From (12), the model (1) with $k_{ij}=y_{ij}-\frac{1}{2}$ can be written as

$$\begin{array}{c} p\left(y_{ij}|\theta_{i},a_{j},b_{j}\right)=2^{-1}\exp\left(k_{ij}\left(a_{j}^{T}\theta_{i}+b_{j}\right)\right)\int_{0}^{\infty }\exp\left(-\frac{1}{2}w_{ij}{\left(a_{j}^{T}\theta_{i}+b_{j}\right)}^{2}\right)p\left(w_{ij}|1,0\right)dw_{ij}. \end{array}$$

Thus, the conditional distribution of $\theta_{i}$ given $y_{i},A,b,w_{i}$ is

(13) $$\begin{array}{cc} p(\theta_{i}|y_{i},A,b,w_{i}) & \propto p\left(\theta_{i}\right)\prod_{j=1}^{J}\exp\left\{k_{ij}\left(a_{j}^{T}\theta_{i}+b_{j}\right)-\frac{1}{2}w_{ij}{\left(a_{j}^{T}\theta_{i}+b_{j}\right)}^{2}\right\}\\ \; & \propto p\left(\theta_{i}\right)\prod_{j=1}^{J}\exp\left\{-\frac{w_{ij}}{2}{\left(a_{j}^{T}\theta_{i}+b_{j}-\frac{k_{ij}}{w_{ij}}\right)}^{2}\right\}\\ \; & \propto p\left(\theta_{i}\right)\exp\left\{-\frac{1}{2}{\left(z_{i}-A\theta_{i}\right)}^{T}\Omega_{i}\left(z_{i}-A\theta_{i}\right)\right\} \end{array}$$

where $z_{i}={\left(\frac{k_{i1}}{w_{i1}}-b_{1},\ldots ,\frac{k_{iJ}}{w_{iJ}}-b_{J}\right)}^{T},\Omega_{i}=\mathrm{diag}\left(w_{i1},\ldots ,w_{iJ}\right)$. In this study, since the prior distribution for $\theta_{i}$ is $N(0,I_{K})$, the conditional distribution (13) becomes a normal distribution, and

(14) $$\begin{array}{c} \theta_{i}|y_{i},A,b,w_{i}∼N\left(\mu_{i},V_{i}\right) \end{array}$$

where $V_{i}={\left(A^{T}\Omega_{i}A+I_{K}\right)}^{-1},\mu_{i}=V_{i}A^{T}\Omega_{i}^{-1}z_{i}$.

Furthermore, considering the conditional distribution of $w_{ij}$, we have

(15) $$\begin{array}{c} w_{ij}|y_{i},a_{j},b_{j},\theta_{i}∼PG\left(1,a_{j}^{T}\theta_{i}+b_{j}\right). \end{array}$$

Therefore, in the t+1th step of the stE-step, we use Gibbs sampling to generate random numbers conforming to the posterior distribution as follows:

(16) $$\begin{array}{cc} {\tilde{w}}_{ij}^{\left(t+1\right)}|y_{i},a_{j}^{\left(t\right)},b_{j}^{\left(t\right)},{\tilde{\theta }}_{i}^{\left(t\right)} & ∼PG\left(1,{\left(a_{j}^{\left(t\right)}\right)}^{T}{\tilde{\theta }}_{i}^{\left(t\right)}+b_{j}\right),\;\left(i=1,\ldots ,N,j=1,\ldots ,J\right) \end{array}$$

(17) $$\begin{array}{cc} {\tilde{\theta }}_{i}^{\left(t+1\right)}|y_{i},A^{\left(t\right)},b^{\left(t\right)},{\tilde{w}}_{i}^{\left(t+1\right)} & ∼N\left(\mu_{i},V_{i}\right),\;\left(i=1,\ldots ,N\right). \end{array}$$

Random numbers from the Pólya-Gamma distribution can be obtained using R packages such as “pg”.

### 3.4. Calculation of Final Estimation Results

In the StEM algorithm, since the stE-step computes stochastically using random numbers as in (9), it does not converge to a certain value like the conventional EM algorithm. Therefore, the final estimation result is obtained by operations such as averaging. In [10], the last *m* steps are used with $\Psi^{\left(t\right)}=\left(A^{\left(t\right)},b^{\left(t\right)}\right)$ as

(18) $$\begin{array}{c} \hat{\Psi }=\frac{1}{m}\sum_{t=T+1}^{T+m}\Psi^{\left(t\right)}. \end{array}$$

However, since stochastic operations are performed in the stE-step, even if regularization terms such as $L_{1}$-regularization or prenet penalty are added, $a_{ij}^{\left(t\right)}$ is not always zero in steps T+1∼T+m. Therefore, if the estimation result is obtained using (18), the advantages of regularization cannot be fully utilized. In this study, for the estimation of A, the median of the last *m* steps is used as

(19) $$\begin{array}{c} {\hat{a}}_{ij}=\mathrm{Med}\left(a_{ij}^{\left(T+1\right)},\ldots ,a_{ij}^{\left(T+m\right)}\right). \end{array}$$

By using the median, if $a_{ij}^{\left(t\right)}$ is mostly zero, ${\hat{a}}_{ij}$ is estimated as zero. Note that for the estimation of b, the average of the last *m* steps is used, similar to (18).

## 4. Numerical Experiments

In this section, we evaluate the performance of the proposed method using synthetic data with a prenet penalty.

### Comparison with Lasso

The synthetic data used in this study, with J=15,K=3, is generated as follows:

$$\begin{array}{c} A={\left(\begin{array}{ccccccccccccccc} 0.4 & 0.7 & 1.0 & 1.3 & 1.6 & 0 & 0 & 0 & 0 & 0 & 0 & 0 & 0 & 0 & 0\\ 0 & 0 & 0 & 0 & 0 & 0.4 & 0.7 & 1.0 & 1.3 & 1.6 & 0 & 0 & 0 & 0 & 0\\ 0 & 0 & 0 & 0 & 0 & 0 & 0 & 0 & 0 & 0 & 0.4 & 0.7 & 1.0 & 1.3 & 1.6 \end{array}\right)}^{T}, \end{array}$$

with b=0. The parameter γ for the prenet penalty is set to 0.1. For lasso, estimation is performed with ρ values of $0.1,0.1\times 0.8^{1},\ldots ,0.1\times 0.8^{20}$, and the BIC is calculated for each. The result with the smallest BIC is chosen as the lasso estimation result. Similarly, for our method with the prenet penalty, estimation is carried out with ρ values of $3,3\times 0.8^{1},\ldots ,3\times 0.8^{20}$, and the result with the smallest BIC is chosen. For both methods, a warm start is performed. The estimation begins by determining the maximum ρ, and subsequently, ρ is decreased. The estimation at each step uses the preceding ρ as the initial value.

First, we evaluate the estimation results of lasso and prenet regularization when data is generated 50 times with N=100. The evaluation metrics used are the mean squared error (MSE) and the correct estimate rate (CER). MSE and CER for the *s*th data’s estimation result ${\hat{A}}^{\left(s\right)}$ can be calculated as follows:

(20) $$\begin{array}{cc} \mathrm{MSE} & =\frac{1}{T}\sum_{s=1}^{T}\frac{∥A-{\hat{A}}^{\left(s\right)}{∥}_{F}^{2}}{JK}, \end{array}$$

(21) $$\begin{array}{cc} \mathrm{CER} & =1-\frac{1}{T}\sum_{s=1}^{T}\sum_{j=1}^{J}\sum_{k=1}^{K}\frac{|I\left(a_{jk}\neq 0\right)-I\left({\hat{a}}_{jk}^{\left(s\right)}\neq 0\right)|}{JK}. \end{array}$$

**Figure 3:**
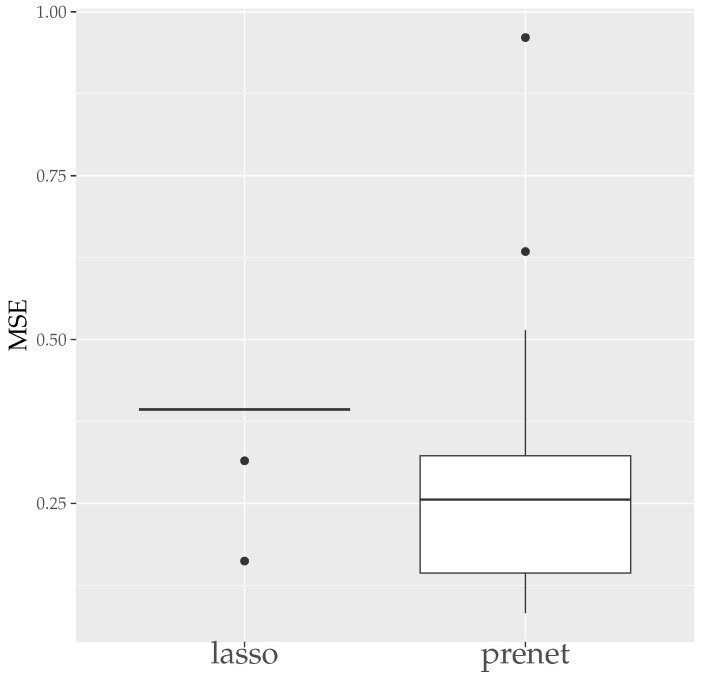
MSE of A (N=100). The median (central line in the box), the interquartile range (width of the box) and the outliers (points outside the lines) are illustrated.

**Figure 4:**
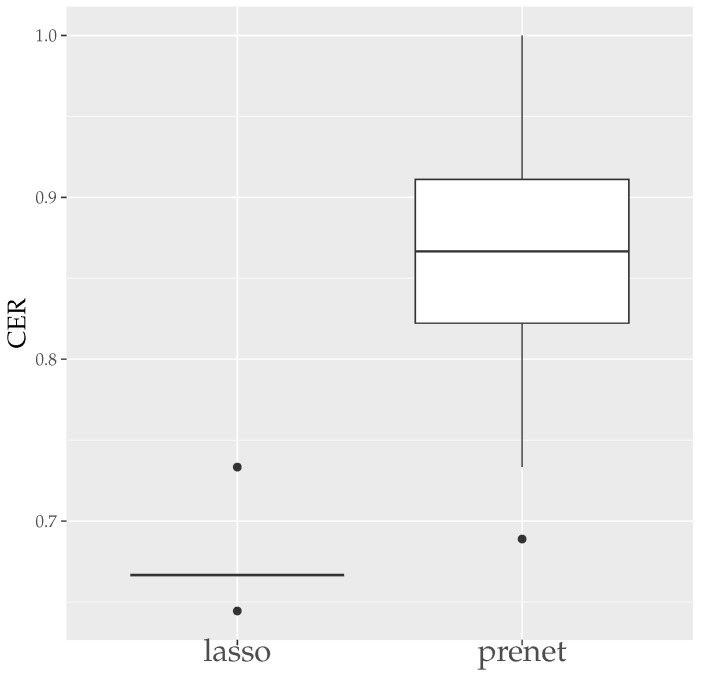
CER of A (N=100). The median (central line in the box), the interquartile range (width of the box) and the outliers (points outside the lines) are illustrated.

MSE measures how well A is estimated, and CER measures how well the structure of A is estimated. Since the estimation results of lasso and prenet are indeterminate with respect to the sign and permutation of the columns of $\hat{A}$, MSE and CER are calculated for all sign and permutation combinations, choosing the smallest one. Figure 3 shows the boxplot of MSE when ρ is selected by BIC. In the case of lasso, choosing ρ by BIC often results in A=O, with A≠O estimated only twice. On the other hand, for prenet, one component in each row does not become zero, resulting in A≠O and generally smaller MSE compared to lasso. Next, Figure 4 shows the boxplot of CER. For lasso, as mentioned earlier, all components become zero, failing to estimate the structure of A well. For prenet, the average CER is around 85%, indicating a good estimation of the structure. As a result, with a small sample size of N=100, lasso fails to estimate the structure of A, reducing all components of A to zero. In contrast, prenet successfully estimates the structure of A, performing better than lasso.

**Figure 5:**
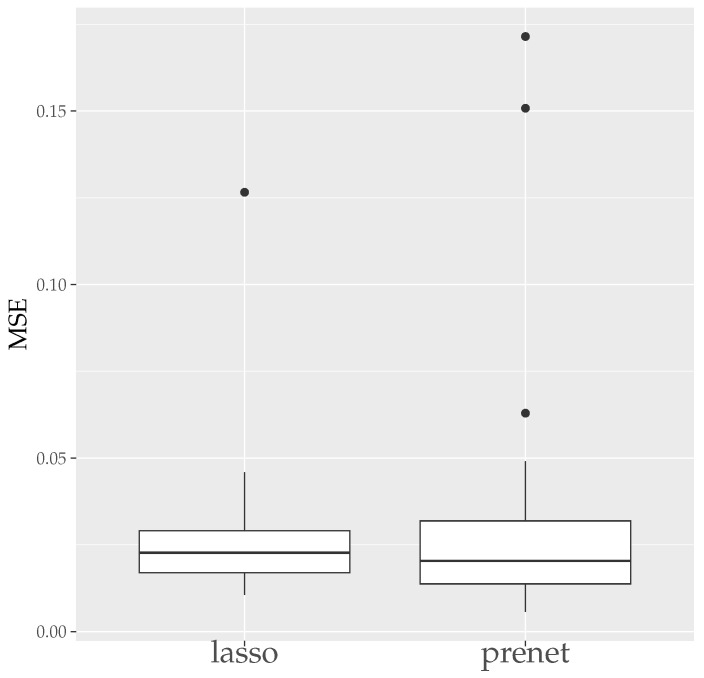
MSE of A (N=500). The median (central line in the box), the interquartile range (width of the box) and the outliers (points outside the lines) are illustrated.

**Figure 6:**
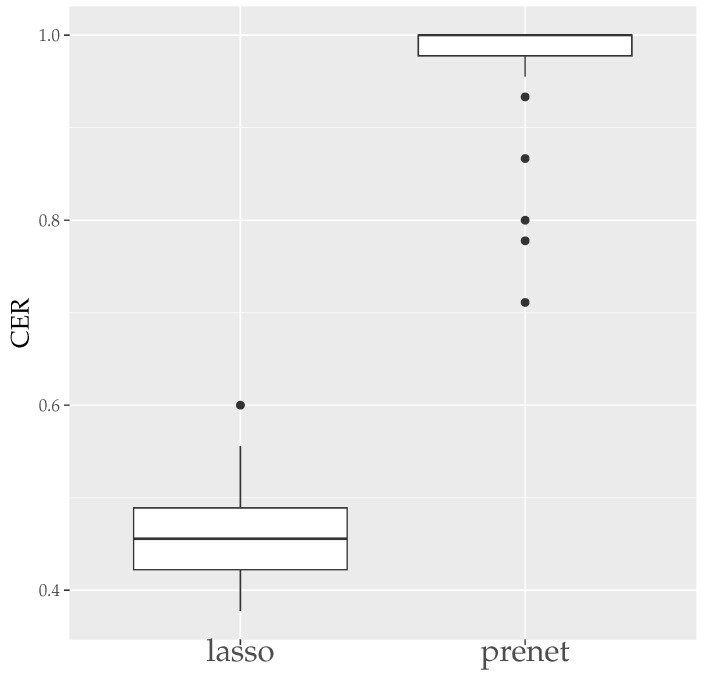
CER of A (N=500). The median (central line in the box), the interquartile range (width of the box) and the outliers (points outside the lines) are illustrated.

Next, we present the boxplots of MER and CER for the estimation results when generating data 50 times with N=500 in Figure 5 and Figure 6. As seen in Figure 5, unlike the case of N=100, the estimation results by lasso are not zero when N=500, and the MSE is smaller compared to prenet. However, the CER of the results of the lasso estimation is low, indicating that it does not accurately estimate the structure of A. On the other hand, prenet has a slightly higher MSE but a higher CER, successfully estimating the structure of A. In fact, in more than half of the cases, the CER reaches 1, perfectly estimating the structure of A.

**Figure 7:**
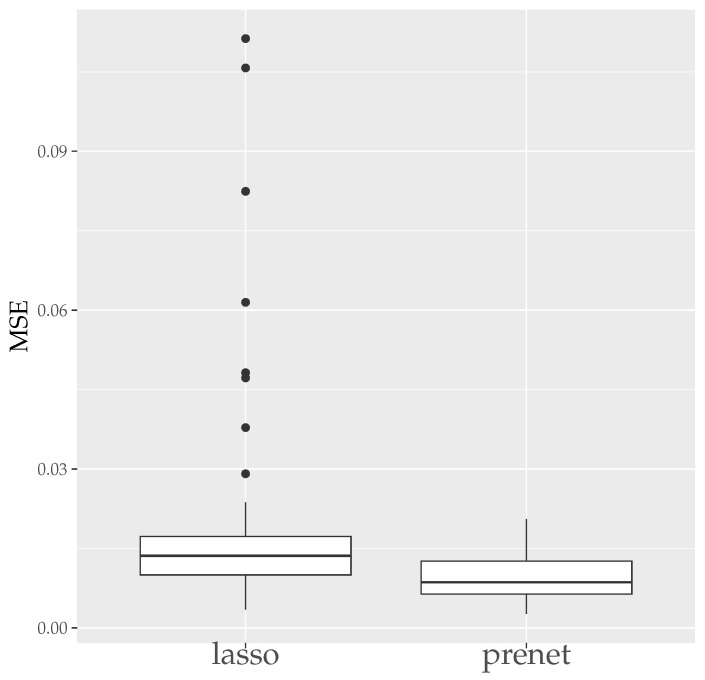
MSE of *A* (N=1000). The median (central line in the box), the interquartile range (width of the box) and the outliers (points outside the lines) are illustrated.

**Figure 8:**
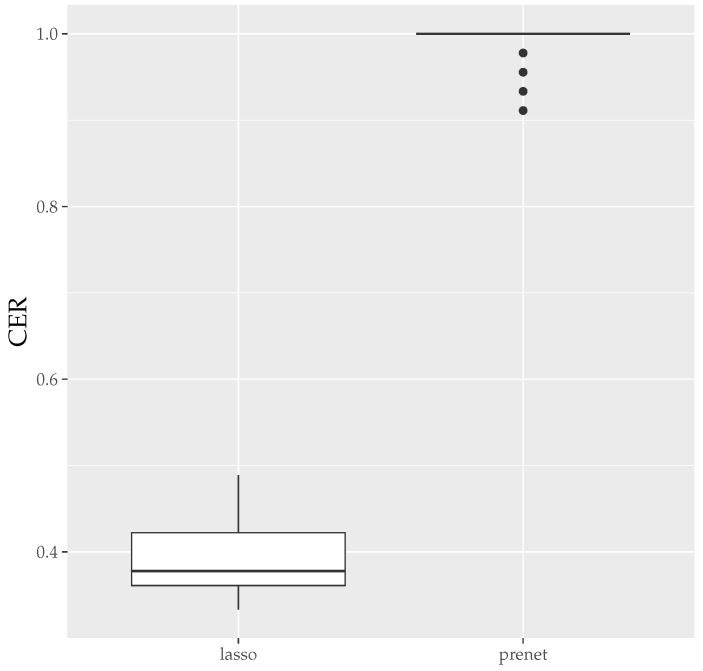
CER of *A* (N=1000). The median (central line in the box), the interquartile range (width of the box) and the outliers (points outside the lines) are illustrated.

Finally, the boxplots of the Mean Error Rate (MER) and the Correct Estimation Rate (CER) for the estimation results generated 50 times with N=1000 are shown in Figure 7 and Figure 8. As seen in Figure 7, unlike the case of N=500, the estimation results by prenet are smaller and more stable compared to lasso when N=1000. Regarding the CER, while lasso is mostly unable to estimate the structure of A, prenet can perfectly estimate the structure of A in most cases, resulting in a CER of 1.

## 5. Conclusions

In this study, we proposed a method for clustering test items in the 2-PL MIRT model using the prenet penalty for structure regularization. The prenet penalty allows each item to be affected by only one latent trait, resulting in a simple structure, making the estimation results easier to interpret. Although the prenet penalty is generally non-convex and thus difficult to optimize directly, it has a multi-convex property where it becomes convex when focusing on one variable and fixing the others, allowing efficient solution via the coordinate descent method. In this study, we efficiently estimated using the stochastic-EM algorithm with the proximal gradient method and the coordinate descent method, also used in lasso. However, the estimation results may stop at a local minimum, so it is necessary to compute several times with different initial values. In numerical experiments, we applied the method to synthetic data and demonstrated that it can estimate the structure of the item matrix better than lasso. With lasso, when the number of subjects is small, using BIC to determine parameters results in all estimates becoming zero, while prenet does not have this issue and can estimate well even with a small number of subjects. Also, with lasso, all estimates shrink, and with large ρ, A=O is estimated, but with prenet, if only one component per row is nonzero, the prenet penalty becomes zero, avoiding such shrinkage. Therefore, the estimates becoming entirely zero is almost non-existent.

In this study, we dealt with the 2-PL MIRT model where responses are binary, but future research should extend to models with guessing and to cases where responses are multi-valued. Also, while this study only dealt with synthetic data, applications to real data and a further detailed evaluation of the performance of the proposed method are necessary. Furthermore, it is necessary to examine how estimates would be affected when imposing additional constraints such as $a_{jk}=0$ for j=1,…,K, k=j+1,…,K−1. 

## Data Availability

The evaluation in the paper is based on synthetic data described above.

## Appendix A. Details of the M-Step

### Appendix A.1. Proximal Gradient Method

Here, we consider the update method in the M-step when the regularization parameters ρ,γ are fixed. The proximal gradient method [11] minimizes a function expressible as f(Ψ)+g(Ψ) with respect to Ψ. Starting with an initial value $\Psi^{\left(0\right)}$ and a step size η>0,

(A1) $$\begin{array}{cc} \Psi^{\left(u+1\right)} & \leftarrow {\mathrm{prox}}_{\eta g}\left(\Psi^{\left(u\right)}-\eta \nabla f\left(\Psi^{\left(u\right)}\right)\right) \end{array}$$

(A2) $$\begin{array}{cc} {\mathrm{prox}}_{\eta g}\left(\Psi^{\prime }\right): & =\underset{\Psi }{\mathrm{argmin}}\{\eta g\left(\Psi \right)+\frac{1}{2}∥\Psi -\Psi^{\prime }{∥}^{2}\} \end{array}$$

is updated in this manner. Here, ${\mathrm{prox}}_{g}$ is called the proximal operator. In the case of this study, to optimize (10) at the t+1 step of the EM algorithm, we set $\Psi =\left(A,b\right)\in R^{J\times K}\times R^{J}$ and

(A3) $$\begin{array}{cc} f(\Psi ): & =-\frac{1}{N}\log p\left(y_{i}|{\tilde{\theta }}_{i}^{\left(t\right)},A,b\right), \end{array}$$

(A4) $$\begin{array}{cc} g(\Psi ): & =\rho P(A). \end{array}$$

Defining $S_{ij}^{\left(u\right)}=P\left(y_{ij}=1|{\tilde{\theta }}_{i}^{\left(t\right)},a_{j}^{\left(u\right)},b_{j}^{\left(u\right)}\right)$, the gradient of *f* is

$$\begin{array}{cc} \nabla_{A}f\left(\Psi \right) & =-\frac{1}{N}{\left(Y-S\right)}^{T}{\tilde{\Theta }}^{\left(t\right)},\\ \nabla_{b}f\left(\Psi \right) & =-\frac{1}{N}{\left(Y-S\right)}^{T}1_{N}. \end{array}$$

Here, ${\tilde{\Theta }}^{\left(t\right)}={\left({\tilde{\theta }}_{1}^{\left(t\right)},\ldots ,{\tilde{\theta }}_{N}^{\left(t\right)}\right)}^{T}$, $Y={\left(y_{1},\ldots ,y_{N}\right)}^{T}$, $S^{\left(u\right)}={\left(S_{ij}^{\left(u\right)}\right)}_{ij}\in R^{N\times J}$, and $1_{N}\in R^{N}$ is a vector with all components as 1. Therefore, applying the proximal gradient method, at each step *u* using the gradients of A,b,

(A5) $$\begin{array}{cc} A^{\left(u+1\right)} & =\underset{A}{\mathrm{argmin}}\left\{\eta P\left(A\right)+\frac{1}{2}{∥A-\left(A^{\left(u\right)}-\eta \nabla_{A}f\left(\Psi^{\left(u\right)}\right)\right)∥}_{F}^{2}\right\}, \end{array}$$

(A6) $$\begin{array}{cc} b^{\left(u+1\right)} & =\underset{b}{\mathrm{argmin}}\left\{\frac{1}{2}{∥b-\left(b^{\left(u\right)}-\eta \nabla_{b}f\left(\Psi^{\left(u\right)}\right)\right)∥}_{2}^{2}\right\} \end{array}$$

are solved to update A,b. Here, ${∥\cdot ∥}_{F}$ is the Frobenius norm, which is the sum of squares of all elements in a matrix.

Since there is no regularization applied to *b*,

$$b^{\left(u+1\right)}=b^{\left(u\right)}-\eta \nabla_{b}f\left(\Psi^{\left(u\right)}\right)$$

can be updated simply. The update method for A is explained in the following section.

### Appendix A.2. Computation of the Proximal Operator

Here, we consider the update of A as in (A5). The prenet penalty is non-convex, making a direct update of A challenging. However, as the prenet penalty is multi-convex, optimizing one variable while fixing the others is straightforward. Therefore, we can solve (A5) using the coordinate descent method.

The calculation formula is as follows:

(A7) $$\begin{array}{cc} {\tilde{a}}_{jk} & =\underset{a_{jk}}{\mathrm{argmin}}\left\{\eta \left(\rho \gamma \sum_{l\neq k}|{\tilde{a}}_{jl}\left|\right|a_{jk}|+\frac{1}{2}\rho \left(1-\gamma \right)\sum_{l\neq k}{\left({\tilde{a}}_{jl}\right)}^{2}{\left(a_{jk}\right)}^{2}\right)+\frac{1}{2}{\left(a_{jk}-\left(a_{jk}^{\left(u\right)}-\eta \frac{\partial }{\partial a_{jk}}f\left(\Psi^{\left(u\right)}\right)\right)\right)}^{2}\right\}\\ \; & =\underset{a_{jk}}{\mathrm{argmin}}\left\{\frac{1}{2}\left(1+\eta \rho \left(1-\gamma \right)\sum_{l\neq k}{\left({\tilde{a}}_{jl}\right)}^{2}\right)a_{jk}^{2}-\left(a_{jk}^{\left(u\right)}-\eta \frac{\partial }{\partial a_{jk}}f\left(\Psi^{\left(u\right)}\right)\right)a_{jk}+\left(\eta \rho \gamma \sum_{l\neq k}\left|{\tilde{a}}_{jl}\right|\right)\right\}\\ \; & =\underset{a_{jk}}{\mathrm{argmin}}\left\{\frac{1}{2}{\left(a_{jk}-\frac{C_{1}}{C_{2}}\right)}^{2}+\frac{C_{3}}{C_{2}}\left|a_{jk}\right|\right\}\\ \; & =\mathrm{sign}\left(\frac{C_{1}}{C_{2}}\right)\times {\left(\left|\frac{C_{1}}{C_{2}}\right|-\frac{C_{3}}{C_{2}}\right)}_{+}\\ C_{1} & =1+\eta \rho \left(1-\gamma \right)\sum_{l\neq k}{\left({\tilde{a}}_{jl}\right)}^{2}\\ C_{2} & =a_{jk}^{\left(u\right)}-\eta \frac{\partial }{\partial a_{jk}}f\left(\Psi^{\left(u\right)}\right)\\ C_{3} & =\eta \rho \gamma \sum_{l\neq k}\left|{\tilde{a}}_{jl}\right| \end{array}$$

Here, $\mathrm{sign}\left(\theta \right)=\left\{\begin{array}{cc} 1 & \theta >0\\ 0 & \theta =0\\ -1 & \theta <0 \end{array}\right.,\theta_{+}=\max\left\{\theta ,0\right\}$. Note that $a_{jk}^{\left(u\right)},\Psi^{\left(u\right)}$ are values from the previous step in the proximal gradient method. Therefore, using (A7), we update for each j,k, and repeat until convergence to find the proximal operator for A.
